# Predicting state level suicide fatalities in the united states with realtime data and machine learning

**DOI:** 10.1038/s44184-023-00045-8

**Published:** 2024-01-16

**Authors:** Devashru Patel, Steven A. Sumner, Daniel Bowen, Marissa Zwald, Ellen Yard, Jing Wang, Royal Law, Kristin Holland, Theresa Nguyen, Gary Mower, Yushiuan Chen, Jenna Iberg Johnson, Megan Jespersen, Elizabeth Mytty, Jennifer M. Lee, Michael Bauer, Eric Caine, Munmun De Choudhury

**Affiliations:** 1 School of Interactive Computing, Georgia Institute of Technology, Atlanta, GA USA; 2 National Center for Injury Prevention and Control, Centers for Disease Control and Prevention, Atlanta, GA USA; 3 Mental Health America, Alexandria, VA USA; 4 Utah Department of Health and Human Services, Salt Lake City, UT USA; 5 Tri-County Health Department, Greenwood Village, CO USA; 6 Louisiana Office of Public Health, New Orleans, LA USA; 7 New York State Department of Health, Albany, NY USA; 8 University of Rochester Medical Center, Rochester, NY USA

**Keywords:** Health services, Public health

## Abstract

Digital trace data and machine learning techniques are increasingly being adopted to predict suicide-related outcomes at the individual level; however, there is also considerable public health need for timely data about suicide trends at the population level. Although significant geographic variation in suicide rates exist by state within the United States, national systems for reporting state suicide trends typically lag by one or more years. We developed and validated a deep learning based approach to utilize real-time, state-level online (Mental Health America web-based depression screenings; Google and YouTube Search Trends), social media (Twitter), and health administrative data (National Syndromic Surveillance Program emergency department visits) to estimate weekly suicide counts in four participating states. Specifically, per state, we built a long short-term memory (LSTM) neural network model to combine signals from the real-time data sources and compared predicted values of suicide deaths from our model to observed values in the same state. Our LSTM model produced accurate estimates of state-specific suicide rates in all four states (percentage error in suicide rate of −2.768% for Utah, −2.823% for Louisiana, −3.449% for New York, and −5.323% for Colorado). Furthermore, our deep learning based approach outperformed current gold-standard baseline autoregressive models that use historical death data alone. We demonstrate an approach to incorporate signals from multiple proxy real-time data sources that can potentially provide more timely estimates of suicide trends at the state level. Timely suicide data at the state level has the potential to improve suicide prevention planning and response tailored to the needs of specific geographic communities.

## Introduction

In the United States (U.S.), there are more than 47,000 suicides annually and suicide rates have increased significantly over the past 20 years^1^. While there are many factors that influence epidemiologic trends related to suicide^2,3^, clear differences exist by geography^4–6^. For example, suicide rates tend to be highest in Western states^7,8^ as well as rural and medium/small metropolitan counties^9^. Changes in suicide rates over time also differ by location^10^.

Robust suicide prevention efforts that are appropriately tailored to specific community needs depend on access to timely information about local epidemiologic trends related to suicide^6^. However, current suicide surveillance approaches are often hampered by a significant lag in reporting suicide deaths. Nationally, lags in the availability of official suicide data reported by the Centers for Disease Control and Prevention (CDC) have historically been several months or more. Although some states possess more timely approaches for reviewing local suicide data, the significant amount of time required to investigate, certify, and report deaths from suicide present a major challenge to public health efforts^11,12^. Importantly, differences in the timeliness of data exist across geography because of differential procedures, technology, or infrastructure available to local county or state medical examiners or coroners^13–15^.

Experts have emphasized the need for using complementary sources of near real-time data to better understand suicide trends^16^. Such data have included large scale online data such as social media, markers of environmental or social risk factors such as economic data, or administrative clinical data^17^, although the study of novel data sources for understanding epidemiologic trends is less developed than the exploration of novel data sources for individual-level or clinical research. For example, Jashinsky et al.^18^ demonstrated that Twitter conversations can be indicative of geography-specific suicide rates; however, the vast majority of the existing research using social media has focused on demonstrating whether social media data may contain signals potentially predictive of individual level suicide-related outcomes (e.g., ideation)^19–27^, and not necessarily how such predictions may assist or augment geographically focused suicide surveillance efforts or conventional public health data^28^. Exceptions include the work of Won et al.^29^, where economic and meteorological data were coupled with social media data to make national-level suicide prediction in South Korea. Moreover, Recently, Choi et al.^30^ combined disparate and real-time data sources in an ensemble machine learning approach to estimate weekly suicide fatalities at the national level in the U.S. with high accuracy. Still, these works do not elucidate how to combine multiple and diverse real-time data sources to nowcast (predicting the present) or forecast (predicting the future) suicide fatalities at sub-national levels and for specific geographic communities^10^.

Despite the near real-time nature of many data sources which may be useful for understanding suicide-related trends, such data sources may suffer from a variety of biases stemming from the fact that these data sources often represent convenience samples^31,32^, the limitations of which may be amplified when used in the context of diverse geographic regions and communities. For instance, although people increasingly use social media and search engines to seek and share health information^33^, including that around mental health and suicide^34^, the amount of use and patterns of use are different across geographic regions^35,36^. Studies have also identified shifting socio-demographic representativeness with other forms of online data, such as that provided by Google trends^37^. Even with health services data drawn from official clinical records, varying access to and utilization of psychiatric services can influence trends across geographic regions^38^. Hence, intelligently and systematically harnessing multiple types of real-time data sources is needed and may provide a more comprehensive estimate of suicide fatalities that spans varied geography, while ameliorating some of the biases and idiosyncrasies of individual data sources.

Consequently, given geographic disparities in suicide rates among U.S. states and the need for more timely suicide data across all states^39^, this study aims to examine whether diverse sources of near real-time information may be leveraged in a machine learning framework to obtain state-specific weekly estimates of suicide fatalities.

## Methods

### Data collection

We used six data sources drawn from both clinical and online sources. These data sources were identified based on prior-literature and theory^33,40–42^, drawing largely on recent research by Choi et al.^30^ that harnessed real-time ensemble data for estimating U.S. national suicide deaths. Because the aim of this prior work was to estimate state-level suicide rates, we considered only data sources available at the state-level. Furthermore, based on data availability, we focused our analysis on four states: Colorado (CO), Louisiana (LA), New York (NY), and Utah (UT). These states provide diverse geographic representativeness to our evaluation and encompass both small, medium, and large population states.

#### Inclusion and ethics statement

Since we utilized secondary administrative data that was either public and/or de-identified, the study did not constitute human subjects research per the ethical review board of the primary and supervisory author’s institution: Georgia Institute of Technology.

#### Online data

We utilized three real-time data sources ascertained from online sources. The three channels were (1) Google search trends (weekly normalized term popularity for 42 suicide- related terms on the Google search engine; 2015–2018); (2) public Twitter data (weekly count of Twitter posts containing 38 suicide-related keywords, phrases, and hashtags; 2015–2018), and (3) YouTube search trends (weekly normalized term popularity for 42 suicide-related terms on YouTube; 2015–2018). Keywords utilized are available in previously published appendices by the authors^30^. The data from the Google and YouTube sources was constrained to the states where the query was made and is made publicly available from the platform as a score from 0 to 100 which represents the normalized popularity of the term over the time period studied. For the Twitter data, we appropriated and expanded a large dataset of public Twitter posts between 2015 and 2018 initially created by Choi et al.^30^, which contained one or more of a set of 38 suicide terms (keywords, phrases, hashtags) identified by a panel of public health experts. Our goal was to assign a geo-location to as many of these postings as possible, based on the location of the author of those same postings—a technique well-established in the social computing literature^43,44^. This was achieved by first utilizing the publicly available self-reported location field of the profile page of all unique authors of the collected posts^45^. Since a user can fill in the location information on their Twitter profile with any text, not necessarily their location, we converted the collected unstructured texts of the profile location fields into a latitude-longitude format by using two popular geocoding APIs, HERE^46^ and OpenStreetMap^47^, which allow inferring the location from a given textual string and provide the inferred location in a standardized geocoded format. Considering only those users whose inferred locations have US state information from either APIs and fall within one of the four states noted above, we labeled the locations of each user as the extracted state information. Finally, we assigned all of the Twitter posts a specific state (out of the four above), based on the state information of their corresponding authors. Then, we aggregated the number of weekly posts for each state in our entire time period of analysis (2015–2018), and used the calculated weekly time-series values as an input for the estimation of suicide fatalities in the corresponding states.

#### Health services data

We next used two data sources which we label as health services data. These consisted of two data sources available in near real-time: (1) the weekly number of emergency department (ED) visits for suicide ideation or attempt provided by each of the four states involved in this research and participating in CDC’s National Syndromic Surveillance Program (NSSP) (Suicide-Related Syndrome, 2015–2018), and (2) weekly averages of self-reported assessments on the Patient Health Questionnaire (PHQ-9), as gathered through continuous online assessments available to the public and administered by the patient advocacy organization Mental Health America (MHA) (2015–2018). Henceforward, these data sources will be referred to as ED and MHA data respectively, and we computed time series signals for both based on aggregation at the state level—location of the EDs and location of the MHA PHQ-9 survey participants as determined by Internet Protocol (IP) address state location.

#### Suicide fatality data

Historical suicide fatality data, aggregated as weekly counts at the state-level, was the primary outcome variable we aimed to estimate. We also utilized lagged historical suicide fatality data as a predictor variable in models. When historical suicide data was used as a predictor variable, only lagged data more than one-year delayed was used in keeping with real-world constraints. Suicide deaths were identified from CDC’s National Vital Statistics System using the International Statistical Classification of Diseases and Related Health Problems, Tenth Revision. The underlying cause of death codes that correspond to such fatalities include U03, X60-X84, and Y87.0.

### Machine learning models

We built and tested a deep-learning based approach to estimate state-level suicide deaths. The choice of the specific methods was motivated from recent advances in deep learning and artificial neural networks in the context of digital health^48^. For all of the approaches described below, aside from the historical suicide fatalities dataset, data in the years 2016, 2017, and 2018 were used for training, validation, and testing respectively.

The modeling approach we pursued used long short-term memory artificial recurrent neural networks (LSTMs)^49^. Unlike standard feed-forward neural networks common in the deep learning field, LSTMs have feedback connections. Consequently, they can not only process single data points (such as documents or images), but also longitudinal sequences of data (such as speech, video, or data streams with temporal relationships—the case in this work)^50,51^. Thus, LSTMs are particularly well-suited to classifying, processing and making predictions based on time series data, since there can be lags of unknown duration between important events in a time series. Furthermore, LSTMs were developed to deal with the vanishing gradient problem that can be encountered when training traditional recurrent neural networks (RNNs). Relative insensitivity to gap length is an advantage of LSTM over RNNs, hidden Markov models, and other sequence learning methods in numerous digital health applications. This motivated our choice of the approach in this work, given the large diversity of near real-time time series data from various data sources.

As the input to the LSTM, separate models were built and evaluated using the individual data sources, as well as their combinations; for instance we developed baseline models with health services data as well as baseline models with online data, for the states under consideration. For each such model, we provided the past two weeks of data, including the current week’s data (for all real time data sources except the historical suicide fatalities) to estimate suicide fatalities in that same week. For instance, to predict the weekly suicide fatalities for a particular state in the first week of a given year, state-level data from the last week of the previous year and the first week of the current year would be used as an input. These sequences of time series values were then adjusted using a sliding-window approach to obtain predictive estimates in subsequent weeks for the corresponding state.

Finally, to tune the optimal hyper-parameters of the LSTM model per state, following standard machine learning procedures, we used a limited grid search procedure of possible values for practical considerations of compute time and resources needed. The parameters we experimented with were (1) number of hidden layers {1, 2}, (2) hidden dimensions {16, 32, 64}, and (3) epochs {150, 200, 250, 300}. Using the combined data stream as the input, each combination of hidden layers, hidden dimensions, and epochs was tested and the performance compared. For the other hyper-parameters, we adopted the default values as follows: Dropout: 0.2 (dropout applied between LSTM layers); Activation Function: Sigmoid for gating mechanisms, Tanh for activation of memory cells; Learning Rate: 0.001; Optimization Algorithm: Adam; Initialization Method: Xavier/Glorot initialization; and Regularization Technique: L2 regularization with a weight decay of 0.01. The best performing combination for any given state was defined as the one that produced the lowest root mean squared error (RMSE). In addition, we used Pearson correlation coefficient to understand the alignment between actual and estimated suicides per week and per model type, and the Mean Absolute Difference metric to assess the extent to which the predicted number of weekly suicide deaths diverged from the actual counts.

In order to evaluate the performance of our models in providing weekly estimates of suicide deaths in the four states under consideration, we additionally built a baseline model for comparison. Motivated from Choi et al.^30^, this baseline model simulates current state-of-the-art forecasting approaches based on using historical suicide fatalities. Autoregressive approaches that harness historical data of the same predictive variable are common in computational social science^52^ and public health research^53^. In order to ensure a fair comparison between the approaches in Section 2.2.1 and the baseline model from a model sophistication perspective, for each state, we built a separate LSTM on the lagged historical suicide fatalities data alone. Prior work has revealed that LSTMs typically improve over autoregressive approaches like autoregressive integrated moving average (ARIMA) in time series forecasting^49^.

In addition to the baseline approach, we compared our deep learning based LSTM approach to other potential leading ensemble modeling approaches. First, we implemented the ensemble approach developed by Choi et al.^30^, which used a two-phase pipeline—the first or intermediate phase fits optimal machine learning models to each individual data stream, while the second phase subsequently combines the predictions made from each data stream via an artificial neural network into a single estimate. We adapted this default model to the estimation task at the state-level for each of the states.

Second, again at the state-level, we considered a single phase estimation pipeline that harnessed all data sources at once in a supervised learning approach and identified optimal features for the learning task using principal component analysis (PCA)^54^. Unlike the ensemble model above which determined the best machine learning model for each data source and then combined these outputs using a neural network, this model approached the problem in a different manner so that the relationships between the data sources could be harnessed. The data was first concatenated into a single vector which included the time series data of the entire training period for the particular state. PCA was then applied to this concatenated vector to reduce dimensions and identify a set of the most representative, non-redundant features. Essentially, rather than relying on the neural network to combine estimates from each data source together, this PCA-based approach combined and meaningfully fused the features across data sources before coming up with the final estimate. The PCA-fused features were then fed into various machine learning models: elastic net, LASSO (least absolute shrinkage and selection operator), linear regression, random forest regression, ridge regression, and support vector regression. The best model, per state, was determined using RMSE.

## Results

### State-wise comparison of performance

Table 1 presents the results of our LSTM model for each of the four states. The selected hyper-parameters for the best models are as follows: the number of hidden layers was 1 for all states; the number of hidden dimensions were 32, 16, 64, 16 for UT, LA, NY, and CO respectively; and the number of epochs were 200, 200, 250, 150 respectively for the same states, UT, LA, NY, and CO. The main metric of interest is the estimated suicide rate per 100,000 population made by the model and the error for this estimate when comparing to actual, observed values. Each of the four states exhibited an error rate of approximately 5 percent or less, for the All Sources model. Specifically, error rates were −2.768% for Utah, −2.823% for Louisiana, −3.449% for New York, and −5.323% for Colorado. The RMSE metric provides a measure of the number of weekly suicide deaths that a particular model’s estimates deviated on average from true values. RMSE ranged from 3.765 in Utah to 7.414 in New York for the All Sources model. In addition, Table 1 also reports the Pearson correlation coefficients between the actual and predicted weekly suicide deaths as well as the Mean Absolute Difference (MAD) metrics; the latter computed as the median of the absolute difference between the actual and predicted values for each model. Correlation was the highest for NY (0.475) and lowest for LA (0.061) when considering the All Sources model. For MAD, the best performance of the All Sources model was for UT (3). These results indicate state-wise differences in model performance, a point we discuss later on in the paper.

**Table 1:** Performance of long short-term memory models for various possible combinations of data sources, by state during the test year 2018.

ASR per 100,000	Source	ESR per 100,000 (Annual Error Rate, %)	RMSE	MAD	Pearson corr.
Colorado (22.51)	Online Data, Combined	20.765 (−7.751%)	5.782	8	0.379
	Health Services, Combined	19.931 (−11.458%)	6.675	13	−0.174
	Baseline with Health Services	19.924 (−11.488%)	6.745	13	−0.152
	Baseline with Online Data	20.572 (−8.608%)	5.934	12	0.310
	Health Services with Online Data	21.234 (−5.669%)	5.711	8	0.338
	**All Sources, Combined**	**21.312 (**−**5.323%)**	**5.889**	11	**0.223**
Louisiana (15.45)	Online Data, Combined	15.188 (−1.695%)	4.064	6	0.196
	Health Services, Combined	14.951 (−3.227%)	4.113	7	0.065
	Baseline with Health Services	14.851 (−3.877%)	4.147	7	−0.022
	Baseline with Online Data	15.157 (−1.897%)	4.130	7	0.043
	Health Services with Online Data	15.344 (−0.687%)	4.039	6	0.259
	**All Sources, Combined**	**15.014 (**−**2.823%)**	**4.156**	7	**0.061**
New York (8.82)	Online Data, Combined	8.852 (0.363%)	7.370	12	0.523
	Health Services, Combined	8.206 (−6.958%)	8.818	15	0.032
	Baseline with Health Services	8.437 (−4.345%)	8.180	14	0.041
	Baseline with Online Data	8.617 (−2.305%)	7.319	9	0.525
	Health Services with Online Data	8.523 (−3.368%)	7.148	8	0.556
	**All Sources, Combined**	**8.516 (**−**3.449%)**	**7.414**	11	**0.475**
Utah (21.04)	Online Data, Combined	20.476 (−2.68%)	3.775	4	−0.030
	Health Services, Combined	20.152 (−4.219%)	3.797	4	−0.024
	Baseline with Health Services	19.515 (−7.249%)	3.907	5	−0.053
	Baseline with Online Data	21.113 (0.345%)	3.777	4	0.000
	Health Services with Online Data	20.902 (−0.654%)	3.689	3	0.312
	**All Sources, Combined**	**20.458 (**−**2.768%)**	**3.765**	3	**0.065**

Online data sources included Google Trends, YouTube Trends, and Twitter. Health services data included emergency department visits from the National Syndromic Surveillance Program and Patient Health Questionnaire (PHQ-9) assessment scores from Mental Health America. Baseline refers to inclusion of historical suicide fatality data in models. *ASR* actual suicide rate, gathered from CDC WONDER’s officially reported crude suicide rate for the year 2018; *ESR* estimated suicide rate, *RMSE* root mean squared error, *MAD* mean absolute difference. MAD gives the median of the absolute difference between actual and estimated suicide deaths per week. Pearson correlation indicates the correlation between weekly estimates from the LSTM model and actual number of deaths during the same week.

Rows in bold indicate the models using a combination of all of the data sources for each state.

**Fig. 1:**
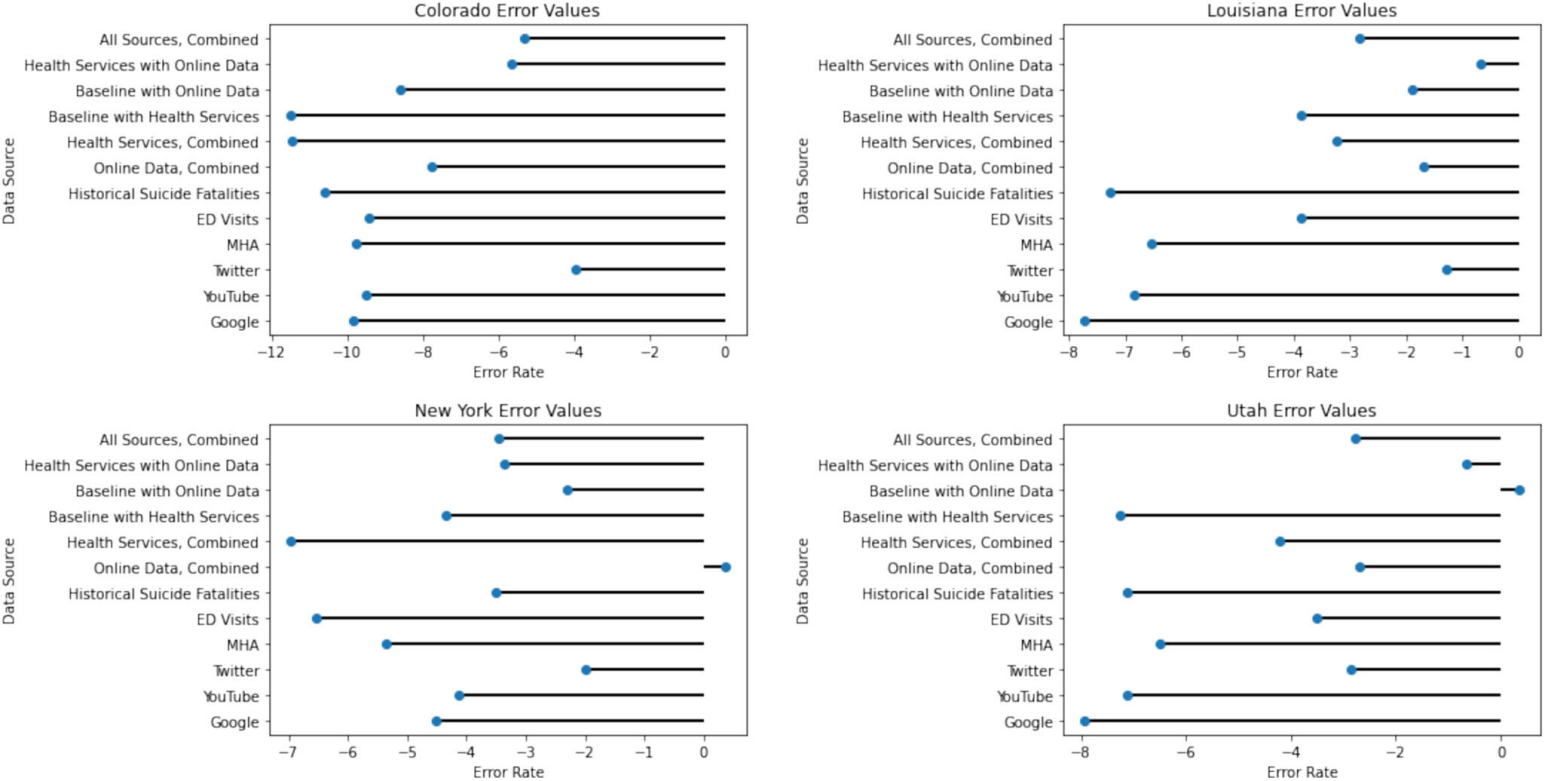
Error rate per data source for all states using long short-term memory models. MHA mental health America, ED emergency department.

To present additional information beyond Table 1, including the performance of individual data sources alone, Fig. 1 plots the percentage errors for each of the individual data sources and key combinations of data sources by state. In general, errors were negative, indicating that models typically predicted suicide counts lower than what actually occurred in 2018. Overall, in all states the LSTM model using all data sources outperformed a model trained on historical suicide fatality data alone (Fig. 1).

Table 2 presents an examination of the error rate, RMSE, MAD, and Pearson correlation for each individual data source for New York, the state with the largest population in our sample and the state with the most suicides. Examining results on this state, we found that the online data sources generally had a lower error rate compared to the health services data sources. Furthermore, the online data sources exhibited a higher week-to-week Pearson correlation.

**Table 2:** Performance of Long short-term memory models using individual data sources for the state of New York.

Category	Source	Estimated suicide rate per 100,000 (annual error rate, %)	RMSE	MAD	Pearson corr.
Online Data	Google	8.422 (−4.511%)	7.849	12	0.382
	YouTube	8.455 (−4.137%)	7.929	13	0.244
	Twitter	8.644 (−1.99%)	7.916	13	0.286
Health Services	MHA	8.348 (−5.352%)	8.730	20	0.037
	ED Visits	8.243 (−6.54%)	8.288	16	−0.240
Baseline	Historical Suicide Fatalities	8.512 (−3.495%)	8.092	14	0.016

*MHA* mental health America, *ED* emergency department, *RMSE* root mean squared error.

### Sensitivity analysis

As a final analysis, Table 3 presents the state-wise results of sensitivity analyses testing alternative methods for combining signals from the data sources. Across the four states, percentage error rates for the annual suicide rate prediction were generally higher for the alternate models considered and more heterogeneous than with the LSTM based models.

**Table 3:** Performance of an RMSE-optimized ensemble model, following Choi et al.^30^ and a PCA based regression model through the concatenation of time series from all data sources.

State	RMSE ensemble		PCA concatenation		
	Pearson	Estimated suicide rate per 100,000 (annual error rate, %)	Pearson corr.	Estimated suicide rate per 100,000 (annual error rate, %)	ASR per 100,000
Colorado	0.271	22.15 (−1.60%)	0.043	20.92 (−7.05%)	22.51
Louisiana	0.234	18.32 (18.58%)	0.180	14.04 (−9.10%)	15.45
New York	0.299	10.57 (19.85%)	0.277	8.40 (−4.76%)	8.82
Utah	0.315	17.61 (−16.30%)	−0.233	20.46 (−2.78%)	21.04

*PCA* principal component analysis, *ASR* actual suicide rate, *RMSE* root mean squared error.

## Discussion

There is considerable public health need for timely state-level data on suicide trends as states can exhibit patterns which deviate from national level trends^10,41^. Furthermore, local level data on suicide is important to facilitate program and policy development specific to a given geographic setting as well as to rapidly detect and respond to abnormal trends, such as those driven by suicide clusters. In this work we developed and validated a modern deep learning based model to estimate weekly suicide fatalities at the state level using multiple proxy real-time data sources. In general, we find good performance with the four states considered here, exhibiting an error of <5% and with the deep learning based LSTM ensemble models outperforming the baseline model that used historical suicide data alone.

We found that models utilizing all data sources typically exhibited improved predictions compared to predictions made from online data, health services data, or historical fatality data alone. The benefit of ensembling or combining signals from multiple disparate data sources was demonstrated in prior national level prediction models and appears to hold for state level models as well^30^. While our models seemingly generalized well, they may not perform well on small population states and with small counts of suicide. Indeed, when examining suicides at the weekly level, it is possible to encounter weeks with zero suicides for states. The presence of low count observations in our time series and general sparsity in suicide deaths at smaller geographic and temporal granularity may have led our models to generally underestimate suicide deaths.

Our findings pave the way for future investigations to examine factors that explain the variability we observed in the ability of different types of real-time data in predicting suicide rates in different states. Regarding the self-reported PHQ-9 data, prior research has observed that depression remains underestimated in the population and this may affect models utilizing data derived from patient screening^38^. Moreover, other scholars have observed that the availability of psychiatric services and allocation of public health funding vary geographically and may be relevant in explaining variations in suicide incidence^55^. Certain types of data, such as ED visit data, might in turn be affected by the availability and extent of access to health services^56^. Future research can test these conjectures with deeper engagement with local data collection efforts.

Finally, Pew Internet Research surveys have persistently revealed systematic socio-demographic (and by corollary, geographic) differences in Internet penetration as well as social media use^57,58^—behaviors that have been found to be highly correlated with both population density and urbanization. Despite being a small selection of U.S. states, our list did include high population density and urban states like NY as well as relatively more sparsely populated/smaller states like UT, or states where the majority of counties are rural, such as LA. The differing performance of our models across states might be explained by these underlying factors. Future research can expand our observations to establish empirical correlates of predictive performance, in relation to metrics like population density and urbanization.

Our study does include some limitations. We note that our approach focused on a limited number of states, limiting the generalizability of both the model as well as the findings. That said, given the diversity of the states in terms of population and socio-demographics, as discussed above, our models still showcase some robustness when applied across differing geographical regions and contexts. We also note that additional data sources (both online and health services) could have been harnessed that might have improved predictive ability of the models – an aspect that is very pertinent to real-world and persistent use of the models for public health efforts, and could be explored in future research. Relatedly, there is some existing research noting the role of environmental factors accounting for regional variations in suicide rates within the same country^59,60^; thus future work could additionally complement the models with such data, although these are not necessarily real-time varying data. Some of the data sources we utilized, such as Twitter data, contained inherent limitations in that information was derived only from public accounts and required geolocation, which introduces some degree of unmeasurable bias. It is interesting to note that our modeling approach, which generally had excellent performance with an approximate error of 5% or less, did consistently underestimate suicide rates. This trend was evident even when utilizing a variety of input data sources and therefore suggests that further improvements to the LSTM model can be made. Furthemore, we speculate that this may have occurred given that suicide deaths nationally had been experiencing a large annual increase over a multiyear period and models may have had difficulty fully capturing the rising rates of suicide in the US. It is also interesting to note, as seen in Fig. 1, that models incorporating all health services and online data sources but excluding historical suicide fatalities generally performed well, sometimes better than models utilizing all data sources including historical suicides. Thus, including historical suicide data itself as a predictor may overindex prediction results to past trends and it should be explored in future research whether utilizing real-time proxy data sources alone yields consistently more reliable estimates. A final limitation relates to the gold-standard for our models – mortality figures reported by local medical examiners and coroners to their state authorities. Rapidly rising rates of opioid-related fatalities during recent years likely has challenged postmortem reviews and suicides may be misclassified as unintentional deaths. How these challenges will influence the future performance of models will require further investigation.

To conclude, the strong performance of our predictive models in estimating state-level weekly suicide fatalities bears significant public health implications. Our models may be particularly useful in times of societal crises, such as the COVID-19 pandemic, to assess differential impacts on suicide in different geographic communities, and thereafter preparing the most suitable response^61^. In general, implementation of programs and policies to prevent suicide^62^ benefits from timely and local data on suicide trends. This work establishes a leading modeling framework for estimating such information to guide suicide prevention efforts.

## CDC disclaimer

The findings and conclusions in this report are those of the authors and do not necessarily represent the official position of the Centers for Disease Control and Prevention.

## Data Availability

The research utilizes public social media data (Twitter), whose sharing in the raw form was restricted by the official API policies of the respective platforms at the time of this paper writing. Google search trends and YouTube data may be requested from the authors subject to appropriate data use agreements. In addition, the paper also uses restricted health data from the CDC, including state-level data on suicide deaths from death certificates and from Emergency Department visits. Due to the nature of the underlying health services data and potentially personally identifiable information, these data are not publicly shareable but can be requested by interested parties through appropriate data use agreements with the respective organizations.

## Code availability

The code for the machine learning algorithm predicting the weekly suicide fatalities in the states of interest can be made available to interested parties. Note that due to the sensitive nature of the data, the code would include untrained predictive models only.
